# Content validity evidence of the Brazilian version of the Cognitive Symptom Checklist-Work-21

**DOI:** 10.1590/0034-7167-2022-0453

**Published:** 2023-09-04

**Authors:** Elaine Cristina Lopes da Rocha, Kayo Henrique Jardel Feitosa Sousa, Paola Alves de Oliveira Lucchesi, Magda Guimarães de Araujo Faria, Marcos Alencar Abaide Balbinotti, Flavio Rebustini, Renata Eloah de Lucena Ferretti-Rebustini, Cristiane Helena Gallasch

**Affiliations:** I Universidade do Estado do Rio de Janeiro. Rio de Janeiro, Rio de Janeiro, Brazil; II Universidade Federal do Rio de Janeiro. Rio de Janeiro, Rio de Janeiro, Brazil; III Université du Québec à Trois-Rivières, Trois-Rivières, Canada; IV Universidade de São Paulo. São Paulo, São Paulo, Brazil

**Keywords:** Validation Study, Cross-Cultural Comparison, Breast Neoplasms, Cancer Survivors, Return to Work, Estudio de Validación, Comparación Transcultural, Neoplasias de la Mama, Supervivientes de Cáncer, Reinserción al Trabajo, Estudos de Validação, Comparação Transcultural, Neoplasias da Mama, Sobreviventes de Câncer, Retorno ao Trabalho

## Abstract

**Objective:**

to cross-culturally adapt and assess the content validity evidence of the Cognitive Symptom Checklist-Work-21 for the Brazilian context.

**Method:**

a psychometric study of cross-cultural adaptation, covering the stages of translation, reconciliation, back-translation, intercultural equivalence assessment and content validity evidence analysis, considering Content Validity Ratio parameters in breast cancer survivors.

**Results:**

the translations were equivalent to the original version. Colloquial expressions were modified, tense, verbal adjusted, and two items containing multiple commands were separated. The final version now contains 22 items, presenting semantic, conceptual, idiomatic and experimental equivalences. The pre-test indicated good understanding and ease in the response process.

**Conclusion:**

the final version was defined as “Lista de verificação de sintomas cognitivos relacionados ao trabalho - 22 itens”, showing good linguistic equivalence and strong evidence of content validity in the Brazilian context.

## RESUMO

**Objetivo:**

realizar a adaptação transcultural e avaliar as evidências de validade de conteúdo do *Cognitive Symptom Checklist-Work-21* para o contexto brasileiro.

**Método:**

estudo psicométrico de adaptação transcultural, abrangendo as etapas de tradução, reconciliação, retrotradução, avaliação de equivalência intercultural e análise das evidências de validade de conteúdo, considerando parâmetros de *Content Validy Ratio* em sobreviventes de câncer de mama.

**Resultados:**

as traduções demonstraram equivalência à versão original. Expressões coloquiais foram modificadas, o tempo verbal ajustado, e dois itens contendo múltiplos comandos foram separados. A versão final passou a conter 22 itens, apresentando equivalências semântica, conceitual, idiomática e experimental. Os valores de CVR mantiveram-se acima de 0,87. O pré-teste indicou boa compreensão e facilidade no processo de resposta.

**Conclusão:**

a versão final foi definida como “Lista de verificação de sintomas cognitivos relacionados ao trabalho - 22 itens”, apresentando boa equivalência linguística e fortes evidências de validade de conteúdo no contexto brasileiro.

## RESUMEN

**Objetivo:**

realizar la adaptación transcultural y evaluar las evidencias de validez de contenido del Cognitive Symptom Checklist-Work-21 para el contexto brasileño.

**Método:**

estudio psicométrico de adaptación transcultural, abarcando las etapas de traducción, reconciliación, retrotraducción, evaluación de equivalencia intercultural y análisis de evidencias de validez de contenido, considerando parámetros de Content Validity Ratio en sobrevivientes de cáncer de mama.

**Resultados:**

las traducciones fueron equivalentes a la versión original. Se modificaron las expresiones coloquiales, se tensaron, se ajustaron las verbales y se separaron dos ítems que contenían múltiples comandos. La versión final ahora contiene 22 ítems, presentando equivalencia semántica, conceptual, idiomática y experimental. El pre-test indicó buena comprensión y facilidad en el proceso de respuesta.

**Conclusión:**

la versión final fue definida como “Lista de verificação de sintomas cognitivos relacionados ao trabalho - 22 itens”, mostrando buena equivalencia lingüística y fuerte evidencia de validez de contenido en el contexto brasileño.

## INTRODUCTION

Cancer has stood out for its high incidence and mortality. Estimates by the Agency for Research on Cancer pointed to 19.3 million new cases of cancer for 2020 and 2.3 million new cases of breast cancer worldwide^(1)^. Brazil follows the high incidence rates of cancer in the world. For the three-year period 2020-22, an estimated 625,000 new cases per year and 66,280 cases of breast cancer^(2)^. In Brazilian women, breast cancer represents the main cause of death. In 2019, 18,068 deaths were computed, equivalent to 16.4% of all cancers that affect this population^(3)^.

Despite the high mortality, there are changes in the approach to cancer with the promotion of practices coordinated by the State, in particular the guidelines for diagnosis and early detection, which allow timely treatment and increased survival^(4-5)^. In the United States (USA), the five-year survival rate for breast cancer is 90.3%^(6)^. In other developed countries, such as Germany, this rate is 87%^(7)^. The increase in the number of post-treatment survivors, combined with the high incidence of breast cancer among women of working age - less than 64 years of age - has guided the debate about return to work for this population group^(8)^.

A study involving 266 cancer survivors showed that 52.6% returned to work without difficulties; 42.5% had some difficulty; and 4.9% were not reinstated, with a reduction in working hours being common^(9-11)^. Among 175 women diagnosed with breast cancer, 87.5% stopped work activities and only 50% returned to work^(12)^. Even more worrying was the finding that, after diagnosis, 39.4% were unemployed and 14.9% of survivors left their jobs within one year^(12-13)^.

The experience of returning to work was considered positive by women who survived breast cancer^(14-15)^. However, it has been reported that health complications derived from treatment sequelae can lead to a recurrence of sick leave, impacting the resumption or continuity of work activities^(16)^.

Return to work after cancer treatment has positive results in terms of self-esteem, sociability, income, source of pleasure and quality of life^(11-12,16-17)^. The psychological impact, cognitive decline, possible physical limitations and concerns about work are considered barriers to this process^(9,11-12,17)^.

Cognitive impairment is an adverse reaction caused by cancer treatment that can cause work-related disabilities and impact the quality of life of these women. The cognitive domains most affected by treatment include learning, memory, executive functions and psychomotor speed. Imaging studies have documented that such impairments are associated with damage to brain structures and changes in functional activity^(18-20)^.

Therefore, in order to improve the work capacity of women survivors of breast cancer, it is necessary to assess and identify possible cognitive impairments in order to offer support and care that allow the rehabilitation of these patients for their reintegration into work activities^(14-15)^.

The Cognitive Symptom Checklist-Work-21 (CSC-W21) is a self-report instrument developed in the USA to screen cognitive symptoms in occupationally active breast cancer survivors, which may represent limitations for returning to work^(21-22)^.

The instrument has 21 items and three dimensions: working memory; executive function; and task completion. The answers to the items are dichotomous in nature, and the final score is presented by the sum of the item scores. The higher the score, the more work-related cognitive limitations the respondent has^(21-22)^.

The CSC-W21 has adequate specificity to identify cognitive symptoms that may impact the work activities of breast cancer survivors, and has already been translated into Chinese and Dutch^(21-23)^. However, there are no cross-cultural adaptation studies or validity evidence assessment of the referred instrument for its use in the Brazilian context.

Considering the relevance of the phenomenon and the lack of tools to identify work-related cognitive limitations in breast cancer survivors in Brazil, this is considered a health measurement instrument relevant to the Brazilian context.

## OBJECTIVE

To cross-culturally adapt and assess the content validity evidence of the CSC-W21 for the Brazilian context.

## METHOD

### Ethical aspects

The study protocol was approved by the Research Ethics Committee of the proposing institution. All participants were informed about their objectives and signed the Informed Consent Form, as provided by Resolution 466/2012.

### Study design, period, and place

This is a psychometric study of cross-cultural adaptation of CSC-W21 to the Brazilian context, carried out between June 2018 and January 2020. The translation steps followed the Patient-Reported Outcomes Measurement Information System (PROMIS) recommendations^(24)^, with support from the definitions by Beaton et al.^(25)^, including: *initial translation*, carried out independently by two native Brazilian bilingual translators, one of whom has knowledge in the health area; *reconciliation*, selection of the most appropriate version of the instrument’s components by a third Brazilian and bilingual translator; *back-translation*, back-translation of the reconciled version by an American translator fluent in Brazilian Portuguese, without knowledge of the original version and the initial translations of the instrument; *back-translation review*, comparison with the original version and sending to the authors of the instrument for assessing discrepancies.

Furthermore, analyzes of linguistic equivalence, content validity evidence and pre-test of the version obtained were carried out.

### Population or sample; inclusion and exclusion criteria

To assess the quality of the translations and content validity evidence, the final version was analyzed, using an electronic form, by five bilingual professionals (a psychologist specializing in cognitive development, a specialist in languages and three nurses - two specialists in psychometrics and occupational health and another in oncology), who assessed semantic, idiomatic, conceptual and experimental equivalences, considering the intercultural context^(25)^.

A total of 30 women participated in the cognitive test (pre-test), who were invited to attend the outpatient clinic during the data collection period. After presenting the research, the women completed the instrument and participated in an individual interview, being asked about their understanding, understanding and difficulties in answering the items.

### Study protocol

To assess the equivalences, experts were asked to indicate whether or not the item remained; if not, a suggestion for adequacy was described^(26)^.

Content validity evidence was assessed by an expert panel composed of six bilingual professionals specializing in oncology and/or psychometrics, from different areas of health training.

Experts assessed each item according to the following indicators: clarity (understandable wording appropriate to the concept); theoretical relevance (item content is or is not indispensable in the target culture for measuring the construct); practical pertinence (representativeness in the underlying construct items)^(27)^; and dimensionality (the distribution of items among the factors reflects the theoretical construction)^(28)^.

All recommendations and decisions were documented and sent to the reconciliation stage translator, who assessed the entire process, identifying problematic items and signaling discrepancies between versions. In the end, the instrument was formatted and revised regarding linguistic and design aspects by the team of researchers, an expert and a translator.

Cognitive testing (pre-test) was performed at the mastology outpatient clinic of a public hospital that is a reference for cancer treatment in the city of Rio de Janeiro-RJ, Brazil, to assess the instrument understandability and applicability to the target population. Patients aged over 18 years, inserted in the labor market before breast cancer diagnosis, formal or informal work, and who had completed chemotherapy treatment for at least two years were included in pre-test. Patients with a history of recurrences or metastases, who were illiterate or who had reading and comprehension difficulties were excluded.

### Analysis of results, and statistics

In linguistic equivalence assessment, items with an agreement index of less than 80% were reviewed^(26)^.

For content validity evidence analysis, for each indicator assessed, the Content Validity Ratio (CVR) was calculated. Items with results below 0.87 were reviewed^(29)^.

In the cognitive testing phase, or pre-test, participants’ comments were analyzed by the researchers to consolidate the Brazilian version of CSC-W21.

## RESULTS

The initial translations, carried out by independent translators, were similar, however they showed small differences in the use of verb tenses and words. In translation 1 (T1), it was suggested that the title be called “*Lista de Conferência de 21 itens de sintomas cognitivos*”. In translation 2 (T2), it was suggested that the word “checklist” be maintained, sustaining the title “*Checklist sobre Sintomas Cognitivos no trabalho - 21 itens*”.

The reconciled version was made by a third translator who chose to use sentences in the first person. This “pre-final” version was back-translated and assessed by the author of the original instrument and experts. In the back-translation stage, minimal discrepancies with the original instrument were evidenced, with no suggestion of changing this version. Chart 1 presents the reconciled and back-translated version of CSCW-21.

**Chart 1:** Reconciled and back-translated versions of CSCW-21, Rio de Janeiro, Brazil, 2020

Original	Reconciled	Back-translation
**Title**		
Cognitive Symptom Checklist- Work-21 item (CSC-W21)	*Lista de conferência sobre sintomas cognitivos no trabalho - 21 itens (CSC-W21)*	Checklist of cognitive symptoms at work - 21 items (CSC-W21)
**Instructions**		
Please read each of the following items below. They describe problems that you may or may not experience at work.	*Por favor, leia cada um dos itens abaixo. Eles descrevem problemas que você pode ou não apresentar durante o seu trabalho.*	Please, read each of the items below. They describe problems that you may or may not have at work.
**Items**		
(Q1) I have difficulty remembering what I intended to write	*Eu tenho dificuldade para lembrar sobre o que eu pretendia escrever*	I have difficulty remembering what I intended to write about
(Q2) I have difficulty remembering my train of thought as I am speaking	*Eu tenho dificuldade para seguir minha linha de raciocínio enquanto estou falando*	I have difficulty following my line of reasoning while I am talking
(Q3) I have difficulty remembering the content of telephone conversations	*Eu tenho dificuldade para lembrar do conteúdo das conversas telefônicas*	I have difficulty remembering the content of telephone conversations
(Q4) I have difficulty remembering the content of conversations and/or meetings	*Eu tenho dificuldade em lembrar do conteúdo das conversas e/ou das reuniões*	I have difficulty remembering the content of conversations and/or meetings
(Q5) I have difficulty remembering a word I wish to say	*Eu tenho dificuldade para lembrar uma palavra que eu gostaria de dizer*	I have difficulty remembering a word I would like to say
(Q6) I have difficulty remembering the name of a familiar object or person	*Eu tenho dificuldade para lembrar o nome de um objeto ou pessoa familiar*	I have difficulty remembering the name of a familiar object or person
(Q7) I have difficulty remembering information that is “on the tip of my tongue”	*Eu tenho dificuldade para lembrar informações que estão na “ponta da minha língua”*	I have difficulty remembering information that is on “the tip of my tongue”
(Q8) I have difficulty remembering things someone has asked me to do	*Eu tenho dificuldade para lembrar de coisas que alguém me pediu para fazer*	I have difficulty remembering things that someone has asked me to do
(Q9) I have difficulty understanding a system	*Eu tenho dificuldade para entender um sistema*	I have difficulty understanding a system
(Q10) I have difficulty understanding how a task fits into a plan or system	*Eu tenho dificuldade para compreender como uma tarefa se encaixa em determinado plano ou sistema*	Eu tenho dificuldade para compreender como uma tarefa se encaixa em determinado plano ou sistema
(Q11) I have difficulty knowing where to look for information to solve a problem	*Eu tenho dificuldade para saber onde buscar informações para resolver um problema*	I have difficulty knowing where to look for information to solve a problem
(Q12) I have difficulty understanding systems and models	*Eu tenho dificuldade para compreender sistemas e modelos*	I have difficulty understanding systems and models
(Q13) I have difficulty figuring out how a decision was reached	*Eu tenho dificuldade em compreender como uma decisão foi alcançada*	I have difficulty understanding how a decision was reached
(Q14) I have difficulty using new information to re-evaluate what I know	*Eu tenho dificuldade em usar novas informações para reavaliar o que eu sei*	I have difficulty using new information to reassess what I know
(Q15) I have difficulty considering all aspects of what I hear and see instead of focusing on only one part	*Eu tenho dificuldade em considerar todos os aspectos sobre os quais ouço e vejo, ao invés de me focar somente em uma parte*	I have difficulty considering all the things I hear about and see, instead of focusing on only one part
(Q16) I have difficulty understanding what a problem is when it occurs and clearly stating what the problem is	*Eu tenho dificuldades em compreender qual é o problema, quando ele o ocorre e em expressar claramente do que se trata*	I have difficulty understanding what the problem is, when it happens and in expressing clearly what is involved
(Q17) I have difficulty following the flow of events	*Eu tenho dificuldade para acompanhar o fluxo de acontecimentos*	I have difficulty following the flow of events
(Q18) I have difficulty understanding graphs and flowcharts	*Eu tenho dificuldade para entender gráficos e fluxogramas*	I have difficulty understanding graphs and flow diagrams
(Q19) I have difficulty completing all the steps of a task or activity	*Eu tenho dificuldade para concluir todos os passos de uma tarefa ou de uma atividade*	I have difficulty completing all the steps in a task or activity
(Q20) I have difficulty staying with a task until completion	*Eu tenho dificuldade em ficar com uma tarefa até que ela seja concluída*	I have difficulty staying with a task until it is completed
(Q21) I have difficulty putting steps in order such that the most important steps are done first	*Eu tenho dificuldade para organizar os passos de uma atividade de forma que as mais importantes sejam realizadas primeiro*	I have difficulty organising the steps of an activity so that the most important ones are done first

In the back-translation review phase, a comparative table was prepared in order to identify discrepancies in the translations and provide clarification to reviewers of the following steps the purpose of the items. The review was also sent to the original author of the instrument for assessment. No relevant discrepancies were identified that would indicate changes to the items.

In the first stage of independent reviews, equivalence analysis showed satisfactory results with a 96.87% agreement rate. Item 6, for semantic and idiomatic equivalence (60% agreement), and item 15, for experimental equivalence (60% agreement), showed results below expectations, and therefore were indicated for modification.

With regard to evidence of content validity, the clarity indicator showed low CVR values for the title, filling instructions and for items 1, 2, 6, 9, 10, 12, 13, 14, 15 and 16. practical relevance indicator showed low CVR values for items 1, 6 and 16, and low CVR value for the theoretical relevance indicator for item 6.

Items 1, 2, 9, 10, 13, 14 and 15 underwent wording adjustments to improve clarity. Items 6 and 16 had multiple commands and were divided into two and three items, respectively. After adjustments, the first consensus version was obtained, with 24 items (C1).

Version C1 was assessed by experts with experience in chemotherapy treatment for breast cancer and cognition. CVR values were once again calculated for content validity evidence analysis. Table 1 shows the final results of the reassessment of CVR values for the Brazilian version.

**Table 1:** Assessment of content validity evidence for the Brazilian version of the Cognitive Symptom Checklist-Work-21 (CSC-W21 - Br), Rio de Janeiro, Brazil, 2019

Questions	Clarity		Practical pertinence		Related dimensionality	Dimensionality		
		Theoretical relevance		Mean CVR		Work memory	Executive function	Task completion
Título	1.0	1.0	1.0	1.0	-	-	-	-
Instruções de preenchimento	1.0	1.0	**0.67**	0.89	-	-	-	-
Item 1	**0.67**	1.0	1.0	0.91	1.0	0.67	-1.0	-0.67
Item 2	1.0	1.0	1.0	1.0	1.0	0	-0.33	-0.66
Item 3	1.0	1.0	1.0	1.0	1.0	0.33	-0.33	-1.0
Item 4	1.0	1.0	1.0	1.0	1.0	0.67	-1.0	-0.66
Item 5	**0.67**	1.0	1.0	0.91	1.0	0.67	-1.0	-1.0
Item 6	**0.67**	1.0	1.0	0.91	1.0	0.67	-0.67	-1.0
Item 7	**0.67**	1.0	1.0	0.91	1.0	0.67	-0.67	-1.0
Item 8	**0.33**	1.0	1.0	0.83	1.0	0.67	-0.67	-1.0
Item 9	1.0	1.0	1.0	1.0	1.0	0.67	-1.0	-0.67
Item 10	**0.33**	**0.67**	**0.67**	**0.67**	1.0	-1.0	0.67	-0.67
Item 11	**0.67**	1.0	1.0	0.91	1.0	-1.0	1.0	-1.0
Item 12	1.0	1.0	1.0	1.0	1.0	-0.67	0.33	-0.67
Item 13	**0.60**	**0.67**	1.0	**0.67**	1.0	-1.0	0.67	-0.33
Item 14	1.0	1.0	1.0	1.0	1.0	-1.0	1.0	-1.0
Item 15	**0.2**	1.0	1.0	1.0	0.8	-0.67	0.2	-0.67
Item 16	1.0	1.0	1.0	1.0	1.0	-1.0	0.33	0
Item 17	**0.33**	1.0	1.0	0.83	1.0	-1.0	0.33	-0.33
Item 18	**0.67**	1.0	1.0	0.91	1.0	-1.0	0.33	-0.33
Item 19	1.0	1.0	1.0	0.9	0.6	-0.33	-0.33	-0.67
Item 20	**0.67**	1.0	1.0	0.91	1.0	-1.0	0.67	-0.33
Item 21	1.0	1.0	1.0	1.0	1.0	-0.67	0.33	-0.67
Item 22	1.0	1.0	1.0	1.0	1.0	-1.0	-0.33	0.33
Item 23	**0.67**	1.0	1.0	0.91	1.0	-1.0	-0.67	0.67
Item 24	1.0	1.0	1.0	1.0	1.0	-1.0	0.67	-0.33

In this round, item 7 was modified to “*eu tenho dificuldade para lembrar o nome de uma pessoa do trabalho*”, while items 10 and 13 had a CVR value below 0.80 and were removed. At the end of this step, an instrument with 22 items divided into three factors was obtained. Version C2 was revised regarding linguistic aspects and formatted considering the design of the original version, being called “*Lista de verificação de sintomas cognitivos relacionados ao trabalho - 22 itens* (CSC-W22)”.

A total of 30 women who had survived breast cancer participated in pre-test, 53.3% white, 33.3% married, 53.3% with high school education and age between 35 and 77 years. At the time of data collection, 73.33% had a formal employment relationship, but only 40% returned to work.

The CSC-W22 was well accepted by the interviewees, and 96.6% of them had a good instrument understanding. Ten women (33.3%) reported difficulty understanding item 19 (“*eu tenho dificuldades para entender gráficos e fluxogramas*”), two (6.6%) did not know the meaning of “graphs” (*gráficos*) and “flowcharts” (*fluxogramas*).

**Figure 1:**
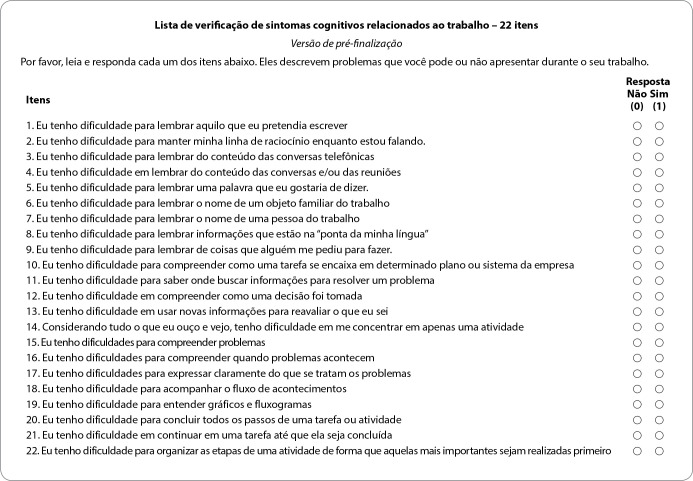
Final version of *Lista de Verificação de Sintomas Cognitivos relacionados ao Trabalho - 22 itens*, Rio de Janeiro, Brazil, 2020

After the pre-test and comment analysis, the Brazilian version of the CSC-W21 was consolidated, which now has 22 items, with three factors as in the original version. Figure 1 shows the final version of “*Lista de Verificação de Sintomas Cognitivos relacionados ao Trabalho - 22 itens*”, obtained after the mentioned steps.

## DISCUSSION

Among breast cancer survivors, work ability is one of the factors that most affect the return to work process. The literature demonstrates that up to 62 determinants may be related to this process, including sociodemographic, professional, financial, clinical, physical and psychological factors, personal values, work environment, personal environment as well as margins of the proposed therapy. Among the late physical and psychological effects derived from cancer treatment, fatigue, concerns about body image, depression or anxiety associated with the fear of recurrence stand out^(30)^.

Cognitive complaints related to memory and executive functions are reported by one in three survivors after oncological treatment, but with unclear physiological mechanisms. Cognitive changes are most often attributed to classical cytotoxic therapies and anti-estrogen treatment that compromise memory, processing speed and cognitive processes performed in the frontal lobe. These manifestations can occur acutely, during treatment, and even in a late phase, being commonly reported among young women, with an important impact on quality of life^(31-32)^.

The process of cross-cultural adaptation (CCA) of assessment instruments is recommended, since health phenomena are culturally determined and socially constructed, and therefore need to be contextualized. Therefore, translations and modifications of CSC-W21 were necessary to propose an instrument capable of identifying, in this conceptual model, the nature of the phenomenon for the Brazilian context. Within a culture, utterances can evoke different meanings due to existing subcultures in society^(33-34)^.

The adoption of an internationally accepted methodological framework enabled the success of the CSC-W21 CCA process for Brazil. Researches have shown that the methodological recommendations of Beaton et al.^(25)^ have been widely used in this type of study, however modifications have been made due to the need for methodological rigor^(34-36)^.

Semantic and cultural issues must be considered in the CCA process of any instrument. In this study, in the translation phase, the main adjustments were related to the adequacy of verbal tenses for the present indicative and modifications of idiomatic expressions to colloquial expressions, mainly avoiding the neologism. Such changes facilitate the understanding of people submitted to these assessments, especially when the instruments are self-administered^(24-25)^.

Back-translation, despite not being unanimous among CCA guidelines, is recommended as an indicator of psychometric evidence quality^(33-37)^. In this study, when compared to the original version, it did not show major discrepancies. Back-translation review by the authors of the original instrument confirmed that the reconciled version did not present item misinterpretations. Thus, this step is considered an important extension of the recommendations by Beaton et al.^(25)^ as it allows for the correction of translation errors that would change the meaning of items.

The independent review stage formed by a multidisciplinary expert committee with extensive clinical experience with women undergoing breast cancer treatment allowed instrument content adaptations that allowed good understanding by the target audience in the cognitive test and ensured applicability to clinical practice^(24-25,38)^.

The assessment by experts allowed us to refine the instrument to a version closer to the original. When assessing the intercultural equivalence, adjustments were related to subtle changes in terms and expressions aiming at greater colloquiality.

The American Educational Research Association establishes that content validity is one of the five validity evidence for health assessment instruments that analyzes the relevance and representativeness of the parts of an instrument for it to achieve its purpose^(39)^. The investigation of validity evidence related to the content of the Brazilian version of CSC-W21 was conducted in stages. In the first, experts’ suggestions were accepted, and the items, modified. In the second stage, changes were validated. Considering the results of this study, the importance of publishing studies assessing this type of evidence and the qualitative process inherent in CCA is reiterated, since it is not possible to adapt an instrument without considering linguistic, semantic and cultural issues^(40)^.

Among the results of the independent review, we highlight the change in the number of items in the instrument due to fragmentation of items 6 and 16 that presented multiple commands. Item writing is one of the most important steps in the instrument development process, as it can affect participants’ responses^(41-42)^. Linguistic formulation is one of the main problems of intercultural equivalence between versions of the same instrument^(33)^. Therefore, the use of multiple commands, intensity adverbs, words with a high emotional charge, unnecessary repetitions and negative, inverted or ambiguous items should be avoided when writing items^(43)^.

The pre-test sample met the literature recommendations^(25)^, and the CSC-W22 (Brazilian version), in general, was well understood by most participants. Only item 19 was less understood, but the analysis of the sample’s profile showed that low education interfered in the conceptualization of “graphs” and “flowcharts”, and not in item wording.

Despite the completion of this study, it is important to carry out further investigations to assess the other evidence of validity, as an internal structure, the relationship with other variables and the consequence of use, in order to provide evidence of instrument validity and reliability for subsequent application to the Brazilian population.

### Study limitations

The limitations of this study are cognitive test application in only one population scenario, which restricts cultural variability in countries like Brazil and lack of screening for cognitive impairment before applying the pre-test.

### Contributions to nursing, health, or public policy

Assessing the cognitive limitations of breast cancer survivors is a necessary task. Providing a valid tool to contribute to improvements in the process of reinserting these women to work can contribute to advances in research and clinical assessments based on good evidence for long-term care planning.

## CONCLUSIONS

The “*Lista de verificação de sintomas cognitivos relacionados ao trabalho - 22 itens*” presented good linguistic equivalence and is culturally equivalent to the “Cognitive Symptom Checklist-Work-21”. Moreover, it gathers strong evidence of content-related validity and was well accepted by the interviewees, being considered an easy-to-understand instrument. However, for its application in the Brazilian context, in order to assess cognitive limitations in breast cancer survivors in order to subsidize the return to work, additional studies to assess other psychometric evidence must be carried out.
